# A Case of Monozygotic Twins: The Value of Discordant Monozygotic Twins in Goldenhar Syndrome—OMIM%164210

**DOI:** 10.1155/2013/591350

**Published:** 2013-08-19

**Authors:** K. N. Venkateshwara Prasad, Arvind Rajha, Pradeep Kumar Vegi

**Affiliations:** ^1^Department of Paediatrics, Sri Devaraj Urs Medical College, Kolar, Karnataka 563101, India; ^2^Scientific Research Laboratory-AHS, Department of Biochemistry, Sri Devaraj Urs Academy of Higher Education and Research, Kolar, Karnataka 563101, India

## Abstract

Goldenhar syndrome is a rare developmental disorder characterised by hemifacial microsomia, epibulbar tumours, ear malformation, and vertebral anomalies. As monozygotic (MZ) twins are believed to be genetically identical, discordance for disease phenotype between MZ twins varies with craniofacial anomalies, cardiac, vertebral, and central nervous system defects sporadically. We report a case of monozygotic female twins discordant for Goldenhar syndrome with hemifacial microsomia and the dysplasia of auricular pinna.

## 1. Introduction

Goldenhar syndrome is one of the rare craniofacial anomalies with incidence of  1 : 3500 to 1 : 5600 births [1]. It is unilateral in 70–80% of the cases [2] as oculoauriculo vertebral (OAV) dysplasia or hemifacial microsomia (OMIM%164210) or facioauriculo vertebral sequence [3]. Nevertheless, the origin of the OAVS is unclear but states as complex and heterogeneous in condition. Two pathophysiologic mechanisms have been proposed for the OAVS: a reduced blood flow and focal hemorrhage in the development region of the first and second branchial arches around 30 to 45 days of pregnancy, in the blastogenesis period. These mechanisms explain the outer ear abnormalities in this spectrum, as the first branchial arch gives rise to the anterior ear primordium and the second branchial arch originates the posterior ear primordium. Also, the outer ear canal derives from the dorsal portion of the first branchial cleft [4]. Although external ear anomalies have been described in OAVS patients—to the point of  being inclusion criteria—middle and especially inner ear alterations have received little attention in the literature [4, 5]. As the function of variability in clinical presentations, there are patients who are afflicted with minimal clinical manifestations, predominantly facial asymmetry and dysplasia of the external ear [6]. This case demonstrates the heterogeneity of spectrums with multifactorial features with frequent alterations. 

## 2. Case Report

 A twenty-year-old 2nd gravida healthy mother delivered female twins per vaginum dated on 19-11-2012, following 36 weeks of uneventful gestation in a rural tertiary care—R L Jalappa Hospital, India. Amongst the twins, the first female twin baby was born by assisted breach delivery and cried soon after the birth with 1.84 kgs by weight, 47 cms in length, and 32 cms head circumference. On examination, the baby was referred as late-preterm baby with an uneventful neonatal period and normal examination. The second female twin baby born by vertex presentation and cried soon after birth; Apgar score at 1 minute was 8/10, weighing 1.94 kgs, 47 cms by length, and 30 cms head circumference. The placenta was completely expelled, monochorionic and diamniotic.

**Figure 1:**
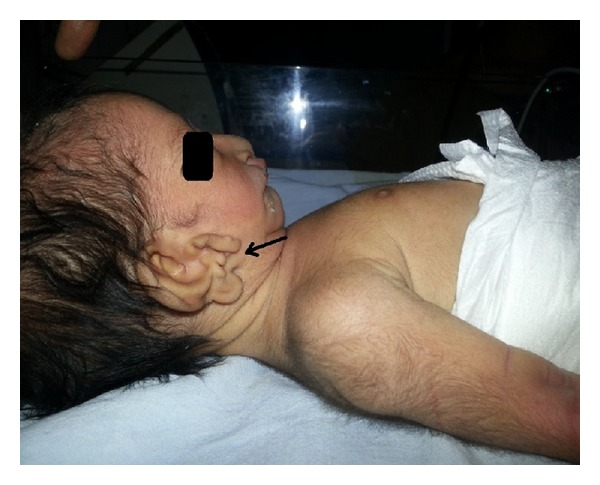
Right ear accessory tags. Right stenosed EAC. Hypoplasia of right malar region. Micrognathia.

**Figure 2:**
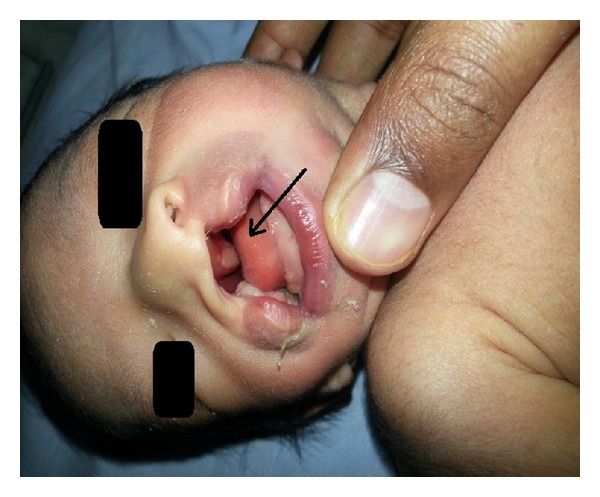
Bilateral complete cleft palate, cleft extending to nasal cavity.

**Figure 3:**
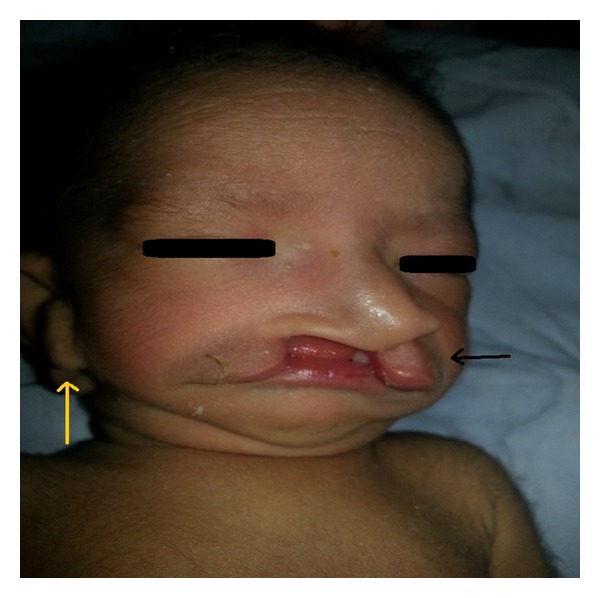
Presenting the right cleft lip, hypertelorism, and micrognathia.

 On clinical examination, the 2nd twin baby showed the following abnormal features as shown in Figures 1 and 2. *Facial features*: hypoplasia of the right malar region, micrognathia, and hypertelorism. *Ears*: bilateral accessory tags, right ear: external auditory canal stenosis. *Oral cavity*: right cleft lip (Figure 3), bilateral complete cleft palate, and right facial cleft (macrostomia). *Nose*: normal external framework and cleft extending to bilateral nasal cavity. There were no skull, spinal, rib, or limb anomalies. Systemic examination did not reveal any cardiovascular or renal abnormality. Investigations revealed normal haemogram levels as follows: Hb 18.4%, WBC count 16800, neutrophils 68%, lymphocytes 20%, and Monocytes 2%, C-reactive proteins −ve, electrolytes: sodium 138 mEq/L, potassium 5.1 meq/L, blood urea −18 mg/dL, and serum creatinine-0.9%. Roentgenograms of the skull, spine, chest, and abdomen were normal. Abdominal ultrasound revealed sonologically normal kidneys.

## 3. Discussion

In the majority of cases, monozygotic twins share a single placenta, and so vascular disruptions are common, which explains the divergent clinical presentation at birth of individuals who are genetically undistinguishable [7]. The etiology of GS is itself related to vascular disruption, predominantly of the stapedial and the external carotid artery, which alters the morphogenesis of structures derived from the first and second branchial arches [8–10]. Most of the cases are sporadic; however, autosomal dominant, recessive, and multifactorial modes of inheritance are also described [1]. Studies postulate that GS is part of a more complex clinical presentation of first and second branchial arches defects and the presence of additional vertebral anomalies with or without epibulbar dermoids [11]. In some of the few cases it is described without structural vertebral anomalies [12]. In most of the cases coloboma of the upper eyelid is observed more frequently. The ear deformities range from preauricular tags of cartilaginous masses to atresia of the external auditory canal, anomalies in the size and shape of the external auricle, and even to anotia. Approximately 10 to 33% of affected individuals are bilateral; the right hemiface is more severely affected than the left hemiface (leading to asymmetry) [3]. 

Anophthalmos, facial palsy, Calcification of falx cerebri, undescended testes, association of GS with Turners syndrome and glaucoma are the rare reported associations. Our case study additionally presented a hemifacial microsomia and the dysplasia of auricular pinna in the monozygotic female twins discordant for GS the rare case. The presentation of the disease and its systemic association varies with age a well established fact. Hence, there is a need, there is a need of multidisciplinary team approach to evaluate all organ systems for anomalies. Pediatric specialists should consult with ear-nose-throat, orthopedics, neurosurgery, and ophthalmology clinicians to decide the most appropriate treatment for the successful outcome.

## 4. Conclusion

 The paucity of GS and its heterogeneity of spectrums demonstrate the multifactorial feature of its pathology. Though GS has a classic triad of ocular, auricular, and vertebral alterations, the clinicians should remember that the hemifacial microsomia and the dysplasia of auricular pinna are more frequent alterations. An early diagnosis of GS should be made which is essential for an appropriate treatment of the affected patients, and care should be taken with genetic counseling.
